# A Case of Multidrug-Resistant (MDR) Tuberculosis and HIV Co-Infection

**DOI:** 10.7759/cureus.37033

**Published:** 2023-04-02

**Authors:** Sebastian G Vila Torres, Jessie Fullmer, Leonard Berkowitz

**Affiliations:** 1 Internal Medicine, The Brooklyn Hospital Center, Brooklyn, USA; 2 Infectious Diseases, The Brooklyn Hospital Center, Brooklyn, USA

**Keywords:** coinfection, multidrug-resistant pathogen, pulmonary tuberculosis, tuberculosis, hiv

## Abstract

Pulmonary tuberculosis (TB) infection is a public health concern in the United States. *Mycobacterium tuberculosis*antimicrobial resistance is a global public health concern. We present a case of a young man from Venezuela who presented to a hospital in New York and was newly diagnosed with pulmonary tuberculosis, human immunodeficiency virus (HIV), and syphilis. His TB isolate was found to be resistant to multiple anti-TB drugs, presenting unusual challenges in treating multidrug-resistant TB with HIV co-infection.

## Introduction

Tuberculosis (TB) caused by *Mycobacterium tuberculosis* remains the number one bacterial cause of death worldwide [1]. In 2021 the World Health Organization (WHO) reported an estimated 10.6 million people fell ill with TB around the world, and 6.7% were people living with HIV [2]. In 2021, 1.6 million people globally died from TB, 11% of whom were coinfected with HIV [3]. In 2021, 7,882 cases were reported in the United States, and an estimated 13 million people live with latent tuberculosis infections [4]. In the Americas, in 2020, there were nearly 300,000 new cases of TB, of which 10% were found to be co-infected with HIV. There were 27,000 TB-related deaths, with 29% having HIV co-infection. Eighty percent of HIV/TB cases are found in seven countries in the Americas: Brazil, Colombia, Dominican Republic, Haiti, Mexico, Peru, and Venezuela [5]. 

While the United States currently has a TB case rate of 2.4/100,000 [4], one of the most drastic increases in the Americas has been in Venezuela. From 2014-2020, the incidence rate rose from 26.6 to 47.0/100,000 [6]. 

TB can be classified by its susceptibility to anti-mycobacterial treatments. Current classifications include drug-susceptible TB (DS-TB), rifampin-resistant (RR-TB), isoniazid, and rifampicin-resistant (MDR-TB), MDR plus resistance to any fluoroquinolone (pre-XDR-TB), and pre-XDR with resistance to any Group A drug (XDR-TB). Group A drugs include levofloxacin, moxifloxacin, bedaquiline, and linezolid [7].

With the ongoing economic crisis in Venezuela, there has been a large migration of these individuals both to neighboring countries and to the United States, which may result in an increased number of cases of patients with TB with varied patterns of resistance as well as TB/HIV co-infection. Since 2015 over 7.1 million Venezuelans have fled the country [8].

## Case presentation

**Figure 1:**
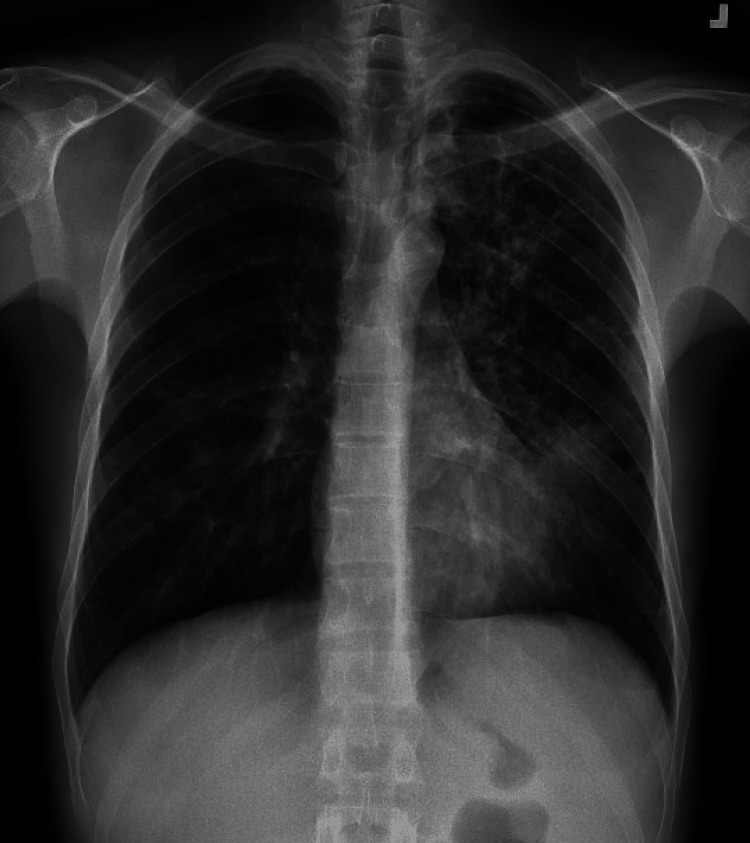
PA view of chest X-Ray PA = posteroanterior

In July 2022, a 30-year-old male with no reported medical history, who had recently emigrated from Venezuela presented to a hospital emergency department in New York City (NYC) with complaints of a productive cough, fever, chills, weight loss, and night sweats for two months. He arrived in the United States on January 2022 and lived in a crowded shelter in NYC. He was unemployed and reported no close contact with known TB-infected individuals. On presentation, he reported no chest pain, shortness of breath, or hemoptysis. He does not take any medications, drink alcohol, or use any illicit drugs; he reported having a history of sexual contact with men. He reported smoking three cigarettes a day for 10 years. On presentation to the emergency department, his temperature was 98.7°F (37°C), blood pressure 95/73 mmHg, heart rate 88 beats per minute, respiratory rate 19 breaths per minute, and oxygen saturation of 99% on room air. A physical exam revealed a cachectic young man with left upper lobe expiratory rhonchi. Chest X-ray showed patchy opacities throughout the left lung, perihilar opacities on the right, and cavities in the left upper lobe (Figure 1).

**Figure 2:**
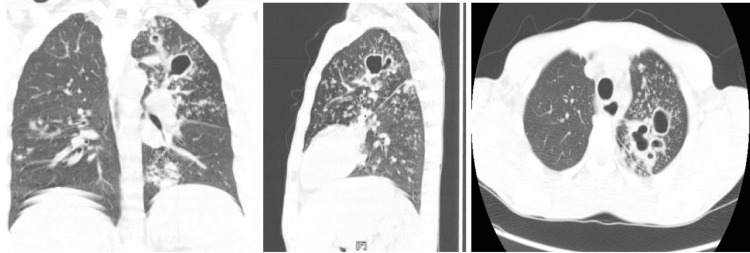
CT of the chest showing left upper lobe cavitations CT = computed tomography

He was admitted and placed in isolation for high clinical suspicion of infection with mycobacterium tuberculosis (TB). He was empirically started on ceftriaxone and azithromycin for the treatment of community-acquired pneumonia. A chest computed tomography (CT) scan without contrast showed left upper lobe cavities with nodular walls and tree-in-bud opacities involving the entire left lung associated with consolidation in the lingula (Figure 2).

He was found to have a positive for acid-fast bacilli (AFB) on a smear test, positive for HIV, and reactive for rapid plasma reagin (RPR). Additional pertinent initial lab work is shown in (Table 1).

**Table 1:** Remarkable lab work TB = Tuberculosis; HIV = Human immunodeficiency virus; RPR = Rapid plasma reagin; CD4: CD-4 T lymphocytes

Labs	On admission	Reference values
White cell count	9.2	4.8-10.8 (K/cm)
Hemoglobin	9.8	13.1-15.5 (g/dL)
Platelet count	312	130-400 (K/cmm)
Sodium	132	136-145 (MMOL/L)
Potassium Serum	4.6	3.5-5.1 (MMOL/L)
Creatinine	0.9	0.7-1.3 (MG/DL)
Glucose random serum	86	60-140 (MG/DL)
Bilirubin total	0.6	0.2-1.2 (MG/DL)
Aspartate aminotransferase	28	8-34 (U/L)
Alanine aminotransferase	34	6-55 (U/L)
Quantiferon TB Gold	Positive	Negative
Sputum acid-fast bacilli smear	Positive	Negative
HIV antigen/antibody	Reactive	Non-reactive
HIV viral load (copies/mL)	71,000	Not detected
HIV genotype	No resistance	No resistance
CD4 cell count (cells/uL)	230	490-1740 cells/uL
RPR	Reactive	Non-reactive
RPR titer	1:32	Negative

Ceftriaxone and azithromycin were discontinued, and he was started on rifabutin, isoniazid (INH), ethambutol, pyrazinamide, and pyridoxine (vitamin B6). Penicillin G benzathine was administered for the treatment of presumptive late latent syphilis as he had no prior history of syphilis, no symptoms, and no prior lab results. HIV anti-retroviral therapy (ART) was initially deferred while TB drugs' tolerability was being assessed and to avoid immune reconstitution inflammatory syndrome (IRIS). Repeat AFB smears while on anti-TB therapy remained positive. The molecular DNA testing of sputum showed the presence of *Mycobacterium tuberculosis* with gene mutations suggestive of rifampin and INH resistance. The New York City Department of Health (DOH) TB division involved in the case recommended discontinuing rifabutin and adding amikacin, levofloxacin, and cycloserine to the treatment plan. After two weeks of well-tolerated anti-TB treatment, the decision to start HIV anti-retroviral therapy was made; hence bictegravir, emtricitabine, and tenofovir alafenamide were added. Repeat AFB smears remained positive after two weeks of starting the new regimen. TB was isolated from the pretreatment sample. The susceptibility testing showed resistance to rifampin, INH, ethambutol, pyrazinamide, but susceptibility to amikacin, cycloserine, and fluoroquinolones. These results were discussed with DOH TB specialists, who recommended switching current treatment to bedaquiline, pretonamid, and linezolid (BPaL) for six-nine months, as per the updated 2020 WHO recommendations for the treatment of drug-resistant tuberculosis [9]. At the time of this writing, the patient has shown clinical improvement. His cough resolved, he gained weight, and his HIV viral load became negative. He had three negative AFB smears and mycobacterial cultures five weeks after starting BPaL therapy. He was eventually discharged with a supply of biktarvy and BPaL. Upon discharge, he reported he was going to live with a friend and agreed to follow up as an outpatient with infectious diseases.

## Discussion

This case highlights the complexity of HIV and pulmonary TB co-infection treatment. The major issues with the treatment of HIV-TB coinfection are drug-drug interactions, risk of immune reconstitution inflammatory syndrome (IRIS), and timing of initiation of ART. In patients who are ART naive antiretroviral therapy should be initiated within two weeks of starting anti-TB medications in patients with CD4 cell counts less than fifty if TB meningitis is not in the differential and within eight weeks for those with higher CD4 cell counts [10]. This patient's clinical stability and CD4 count above two hundred allowed us to start first-line anti-TB drugs, adjust treatment based on drug susceptibilities, and start ART, avoiding significant drug-drug interactions. Although the optimal timing of ART initiation in tuberculosis infection remains controversial, ART should not be delayed until treatment is finished, as ART initiation during TB treatment significantly improves survival [10]. In this case, ART was initiated two weeks after anti-TB drugs were initiated and well tolerated.

The strain of TB isolated in this case showed multidrug resistance. Patients with HIV and MDR-TB co-infection have a fourfold higher mortality risk than MDR-TB infection alone [11]. A recent report from Conradie et al., in patients with MDR TB (not HIV coinfected), showed favorable outcomes with an all-oral anti-TB regimen consisting of bedaquiline, pretomanid, and linezolid for a duration of six months [12]. The results of this study, along with our patient’s rapid viral suppression and a fairly good CD4 count should increase the chances of a favorable outcome.

## Conclusions

The importance of evaluating the whole clinical picture when making treatment decisions is evident in this case. The treatment of TB and HIV co-infection is complicated by major drug-drug interactions and, in this case, further complicated his highly resistant strain of TB; however, the fact that this patient was clinically stable when he presented to the hospital gave us the flexibility to start treatment for TB prior to antiretroviral therapy, preventing potential drug-drug interactions. Once the initial bacterial culture sensitivities became available, the optimal anti-TB treatment was evident, and the best antiretroviral therapy regimen was very clear, leading to effective treatment of the TB and HIV co-infection.
